# Cardiotoxicity evaluation using magnetic resonance imaging in breast Cancer patients (CareBest): study protocol for a prospective trial

**DOI:** 10.1186/s12872-020-01497-y

**Published:** 2020-06-03

**Authors:** Yoo Jin Hong, Gun Min Kim, Kyunghwa Han, Pan Ki Kim, Su An Lee, Eunkyung An, Ji Yeon Lee, Hye-Jeong Lee, Jin Hur, Young Jin Kim, Min Jung Kim, Byoung Wook Choi

**Affiliations:** 1 Department of Radiology and Research Institute of Radiological Science, Severance Hospital, Yonsei University College of Medicine, 50 Yonsei-ro, Seodaemun-gu, Seoul, 120-752 South Korea; 2 Department of Internal Medicine, Yonsei University College of Medicine, Seoul, South Korea

**Keywords:** Magnetic resonance imaging, Cardiotoxicity, Native T1, Extracellular volume fraction, T1 mapping

## Abstract

**Background:**

Cardiovascular disease is second only to cancer recurrence as a determinant of lifespan in cancer survivors, and cancer therapy-related cardiac dysfunction is a clinically important risk factor. We aim to investigate the use of cardiac magnetic resonance imaging (MRI) to evaluate early tissue changes and perform functional assessment of chemo- and radiation-induced cardiotoxicity and to identify MRI prognostic indicators of cardiotoxicity in breast cancer patients.

**Methods:**

A 3-min cardiac imaging protocol will be added to the breast MRI examination to diagnose cardiotoxicity in breast cancer patients. Standardized MRI-based evaluation of breast cancer and the left ventricular myocardium will be performed at baseline and at 3, 6, and 12 months and 2 years or more after cancer treatment. We will analyze both ventricular volume and ejection fraction (EF), strain of left ventricle (LV), native T1, extracellular volume fraction (ECV), and T2 values acquired in the mid LV.

**Discussion:**

The primary result of this study will be the comparison of the prognostic value of MRI parameters (native T1, ECV, both ventricular systolic function and LV strain) for cardiotoxicity. The endpoint is defined as the occurrence of a major adverse cardiac event (MACE). The secondary outcome will be an assessment of the temporal relationships between contractile dysfunction and microstructural injury over 4 years using MRI. This study will assess the usefulness of quantitative MRI to diagnose cardiotoxicity and will clarify the temporal relationships between contractile dysfunction and microstructural injury of the LV myocardium using MRI during breast cancer treatment.

**Trial registration:**

The protocol was registered at clinicaltrials.gov (Clinical trial no. NCT03301389) on October 4, 2017.

## Background

The side effects of cancer therapy have recently attracted significant attention [1]. Earlier diagnosis and improved systemic therapies, including targeted therapies, lead to longer life expectancy after cancer treatment [2–4]. Treatment-related comorbidities have thus become an important issue for long-term cancer survivors [5].

Cancer therapy-related cardiac dysfunction (CTRCD) is the second leading cause of death in cancer survivors after cancer-related mortality [1, 5, 6]. In particular, CTRCD is an important problem in the context of breast cancer because the incidence in young women is increasing while the prognosis is improving due to the development of various new treatments. Moreover, various breast cancer treatments specifically lead to cardiac dysfunction.

Current guidelines consider left ventricular ejection fraction (LVEF) assessment using echocardiography as the standard diagnostic technique for detecting chemotherapy-induced cardiotoxicity [7, 8]. However, magnetic resonance imaging (MRI) may play an important role in cardiac evaluation among cancer patients. MRI is the gold standard for the evaluation of ventricular volumes and function, with greater intra- and inter-observer reproducibility than other modalities, and MRI may achieve higher sensitivity in identifying cardiomyopathy than other diagnostic techniques [8, 9].

The increasingly used T1 mapping sequences improved the usefulness of cardiac MRI, which enables tissue characterization through quantitative analysis of phenomena such as myocardial edema, inflammation, and fibrosis, thus playing an important role in the diagnosis of early and late cardiotoxicity in cancer patients [10, 11]. However, the relationship between contractile dysfunction and microstructural injury is poorly understood. A previous study based on experiments on animals reported that T1-mapping MRI is useful for the early diagnosis of chemotherapy-induced cardiotoxicity [12].

The objective of our proposed study is to investigate the use of cardiac MRI to evaluate early tissue changes and perform functional assessment in chemo- and radiation-induced cardiotoxicity and to identify cardiotoxicity prognostic factors in patients with breast cancer.

### Primary hypothesis

The **Car**diac Magnetic Resonance for **E**arly Detection of Cardiotoxicity in **B**r**e**a**st** Cancer (CareBest) **study** hypothesizes that the evaluation of early tissue changes and functional assessment using cardiac MRI would be useful in the early detection and risk stratification of chemotherapy- and radiation therapy-induced cardiotoxicity in patients with breast cancer.

## This hypothesis is supported by the following considerations

1. In the early phase of exposure to an anthracycline agent, tissue injury and concurrent elevation of native T1 and extracellular volume fraction (ECV) values were noted in the preclinical study, without significant left ventricular (LV) reduction. Therefore, T1 mapping MRI allows for earlier detection of chemotherapy-induced cardiotoxicity [12]. T1 mapping parameters represent good surveillance markers of tissue injury during the early phase of cardiotoxicity.
2. Many studies suggest that CTRCD is not a functional but a structural disorder. The relationship between contractile dysfunction and microstructural injury, such as myocyte damage, fibrosis, and inflammation, remains poorly understood [11]. It was thus hypothesized that the evaluation of tissue changes using cardiac MRI would be more effective for cardiotoxicity risk stratification than the evaluation of functional changes.

## Methods and design

### Overall study design

**Fig. 1:**
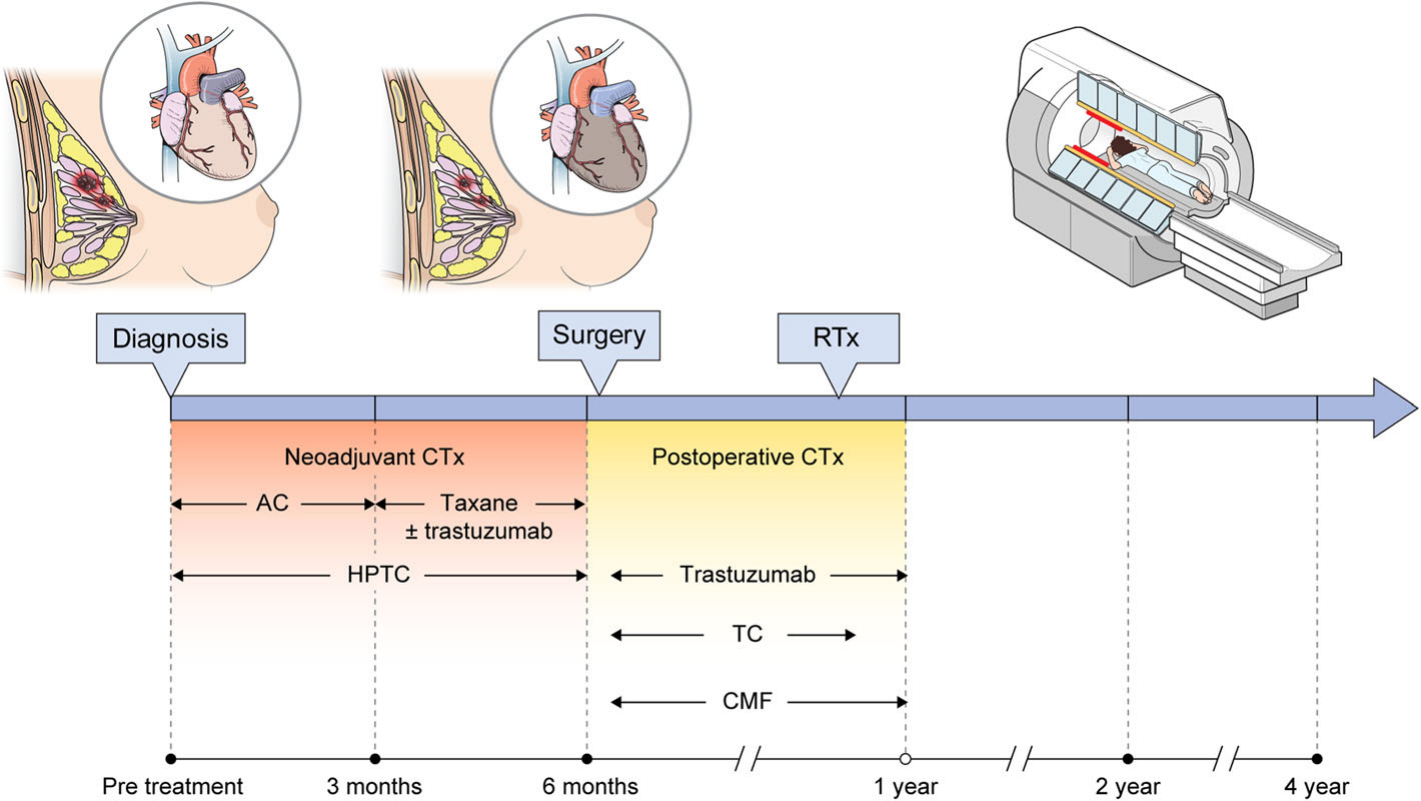
Study design Black circles indicate MRI scanning

**CareBest** is a single-center, large-scale prospective study. To achieve the goal of the study, cardiac MRI protocols for cardiotoxicity diagnosis will be applied to breast cancer patients who have received or are planning to receive chemotherapy. A 3-min cardiac imaging protocol was added to the breast MRI scanning protocol as a screening tool. The protocol includes T1 and T2 mapping and cine imaging of the LV myocardium. MRI is performed at 3 months, 6 months, and 2 years after treatment. Treatment protocols and MRI timing are shown in Fig. 1.

### Primary and secondary objectives

The primary objective of this study is to investigate and compare the prognostic value of cardiac MRI parameters (native and postcontrast T1 mapping, ECV, right ventricular (RV), LV systolic function, and global longitudinal strain of LV) for cardiotoxicity. The endpoint is MACEs.

The secondary objectives are to determine the temporal relationships between contractile dysfunction and microstructural injury over a four-year period using cardiac MRI across all patients and by subgroup analysis according to treatment (e.g., chemotherapy regimen, targeted therapy agents, and radiation dose) and to compare the diagnostic accuracy of MRI, echocardiography or multigated acquisition (MUGA) by subgroup analysis.

### Study population

This prospective study will enroll over 2000 participants referred for breast cancer evaluation using MRI in a single center. All enrolled patients will undergo standardized breast MRI including a cardiac imaging protocol at the time points specified above and blood analysis to assess hematocrit (Hct) levels immediately before each MRI examination.

### Inclusion criteria

Patients ≥18 years of age are considered eligible if, following the initial oncology consultation, breast cancer is diagnosed and related therapeutic plans are formulated. All subjects will agree to participate by giving both verbal and written informed consent. This prospective study was approved by the institutional review board of Yonsei University College of Medicine.

Exclusion criteria include prior mammoplasty, history of other cancers, confirmed benign breast disease, poor image quality due to uncontrolled breast holding, arrhythmia, or patients with contraindications to MRI (i.e., claustrophobia, estimated glomerular filtration rate (eGFR) < 30).

### Cardiac MRI protocol

A standardized breast and cardiac MR examination will be performed with 3 T MR scanners with an 8-channel breast coil (GE MRI 750w, GE Healthcare, Milwaukee, WI, USA). Because breast MRI is performed with the patient in a prone position, peripheral pulse gating will be applied for cardiac imaging.

The cardiac protocol includes pre- and postcontrast T1 mapping, T2 mapping, and cine imaging of both ventricular myocardia. Motion-corrected native T1 and postcontrast T1 and T2 mapping images will be acquired at the end of diastole in the short axis orientation of mid LV using a FIESTA-based 3(3)5 MOLLI sequence. Post-contrast T1 images will be acquired 15 min after the bolus injection of the contrast agent (0.1 mmol/kg body weight of gadolinium-based contrast agent, Gadobutrol 604.76 mg/ml).

The imaging parameters for the 2D FIESTA cardiac cine MRI will be set as follows: flip angle = 45°, repetition time (TR) = 3.0 ms, echo time (TE) = 1.1 ms, field of view (FOV) = 390 × 390 mm, matrix = 160 × 192, measured pixel size = 2.4 × 2.0 mm, slice thickness = 8 mm (2 mm gap between adjacent slices), number of slices = 9, temporal resolution = 60 ms, and ASSET factor = 2.

The specific scan parameters for T1 mapping will be set as follows: flip angle = 35°, TR = 2.8 ms, TE = 0.9 ms, FOV = 300 × 300 mm, matrix = 160 × 128, delay time (TD) = 220 ms, measured pixel size = 1.9 × 2.3 mm, slice thickness = 8 mm, and acquisition window = 361 ms.

The specific scan parameters for FSE-based black blood T2 mapping will be set as follows: TR = 1 RR; total echo train length (ETL) = 16; FOV = 300 × 300 mm; matrix = 160 × 128; measured pixel size = 1.9 × 2.3 mm; slice thickness = 8 mm; 4 echoes with effect TEs = 11.3, 33.9, 56.5, and 79.1 ms; acquisition window = 91 ms; and ASSET factor = 2.

Sequential short axis cine imaging will be performed including the entire ventricular myocardium in addition to standard long-axis views using the 2D FIESTA sequence. The specific scan parameters will be set as follows: flip angle = 45°, TR = 3.0 ms, TE = 1.1 ms, FOV = 390 × 390 mm, matrix = 160 × 192, measured pixel size = 2.4 × 2.0 mm, slice thickness = 8 mm (2 mm gap between adjacent slices), number of slices = 9, temporal resolution = 60 ms, and ASSET factor = 2.

The sequences for breast MR examination include a three-plane localizing sequence, axial T2-weighted fast-spin- echo and T2-stimulated inversion recovery (STIR) sequence, and diffusion-weighted imaging before contrast administration. 3D dynamic postcontrast-enhanced (DCE) images and a T1-weighted 3D delayed postcontrast sequence will be acquired in the sagittal plane after contrast injection.

Native T1 and T2 images will be acquired between T2-STIR and diffusion images, while postcontrast T1 images will be acquired after delayed postcontrast images. Cine images will be acquired before dynamic images. Precontrast T1 and T2 mapping images will be acquired before DWI imaging, and postcontrast T1 mapping imaging will be performed after dynamic contrast imaging. The hematocrit (Hct) levels of the patients will be acquired on the day of cardiac MRI.

### Cardiac MRI analysis

#### Both ventricular volumes and systolic function

All MR images will be analyzed using cvi^42^ image analysis software (Circle Cardiovascular Imaging Inc., Calgary, AB, Canada). Short-axis cine images will be analyzed using semi-automated contouring of the endocardial and epicardial borders of both ventricles at end-diastole and end-systole to calculate both ventricular function (i.e., RV, LV end-diastolic volume (RVEDV, LVEDV), end-systolic volume (RVESV, LVESV), cardiac output, stroke volume, RVEF, and LVEF).

#### T1 and T2 mapping analysis

One native T1, one postcontrast T1, and one T2 mapping image acquired on the mid LV will be transferred to the software. A region of interest (ROI) will be freely drawn on the septum. Images with severe artifacts will be coded and removed from the analysis.

Mean segmental native T1, T2, and postcontrast T1 values are calculated for each patient. ECV values are calculated using the mean values of native and postcontrast T1 and hematocrit, as previously established [13].

#### Myocardial strain analysis

Feature tracking analysis will also be performed using cvi42 software. The long axis and short-axis cine images will be loaded onto the software. Endocardial and epicardial borders of the LV in both the long- and short-axis views were semi-automatically delineated in the end-diastolic phase. The feature software automatically measured the global longitudinal strain in the two-dimensional (2D) longitudinal directions. Analysis of interobserver agreement in regard to MRI measurements will be performed.

### Chemotherapy regimen

#### Cytotoxic chemotherapy

- AC regimen: doxorubicin 60 mg/m^2^ with cyclophosphamide (600 mg/m^2^) IV every 3 weeks for a total of 4 cycles (cumulative dose of doxorubicin: 240 mg/m^2^)
- AC followed by taxane: 4 cycles of AC followed by taxane (paclitaxel 80 mg/m^2^ IV once a week for 12 weeks, or docetaxel 75 mg/m^2^ IV every 3 weeks, for a total of 4 cycles)
- CMF regimen: cyclophosphamide (600 mg/m^2^) with methotrexate (60 mg/m^2^) and fluorouracil (600 mg/m^2^) at days 1 and 8 every 4 weeks, for a total of 6 cycles
- TC regimen: docetaxel 75 mg/m^2^ and cyclophosphamide 600 mg/m^2^ IV every 3 weeks for a total of 4 cycles

#### HER2-targeted therapy

-HPTC regimen: Trastuzumab 8 mg/kg loading dose and 6 mg/kg maintenance dose, pertuzumab 840 mg loading dose and 420 mg maintenance dose, docetaxel 75 mg/m^2^, carboplatin every 3 weeks for a total of 6 cycles.

-Trastuzumab: trastuzumab 8 mg/kg IV (loading dose) followed by 6 mg/kg IV (maintenance dose) every 3 weeks for a total of 18 cycles or trastuzumab 600 mg SC every 3 weeks for a total of 18 cycles for HER2-positive breast cancer: Trastuzumab will be given after the completion of the doxorubicin regimen regardless of the taxane regimen.

### Clinical outcomes

The primary outcome is MACEs defined as CV death, hospitalization due to heart failure, and/or heart transplantation. The secondary outcome is left ventricular dysfunction, defined as a decrease in the LVEF of > 10 percentage points to a value < 53% [9]. The follow-up duration will be up to 4 years after treatment.

### Sample size considerations

There is no generally accepted approach to estimate the sample size for studies of risk prediction models [14]. We will enroll the largest possible number of patients to ensure the stability of the prediction model based on MRI parameters.

## Preliminary study

### Reliability of cardiac imaging during breast MRI

We will perform a preliminary study to evaluate the feasibility of cardiac imaging during breast MRI in control subjects (*n* = 15). All patients will undergo planned cardiac imaging with a cardiac coil in a supine position, without contrast agent. A native T1 cardiac image in a short axis plane will be acquired. After cardiac imaging, the planned breast+cardiac imaging protocol will be performed in a prone position with contrast agent.

## Statistical analysis

Categorical baseline characteristics will be expressed as numbers and percentages, and continuous variables will be expressed as the means and standard deviations. To evaluate the prognostic value of T1 mapping parameters for cardiotoxicity, Cox proportional hazard regression or other transformations will be used after checking the proportional hazard assumption. To determine and compare the prognostic value of native T1 and ECV and that of LVEF or global longitudinal strain of LV, time-dependent ROC curves, Harrell’s C statistic and bootstrapping with 1000 repetitions will be used. A linear mixed model will be used to evaluate the temporal relationship between LVEF, native T1, ECV, and T2 values. The diagnostic accuracies of MRI, MUGA, and echocardiography for cardiotoxicity will be evaluated by ROC curves, and the significance of the differences among the related areas under the curves (AUC) will be evaluated using Delong’s method [15]. *P*-values less than 0.05 will be considered statistically significant. All statistical analyses will be performed using SAS (version 9.4, SAS Institute Inc., Cary, NC, USA).

### Study approval and progression

The study protocol was approved by the regional institutional review board in October 2016 (IRB No. 4–2016-0730), and the protocol is registered at clinicaltrials.gov (Clinical trial no. NCT03301389). A data and safety monitoring board was constituted to ensure the safe continuation of the study.

## Discussion

This prospective study will be the largest study investigating serial MRI-based evaluation of CTRCD. We will investigate the effects of chemotherapy, radiation therapy, and other therapies on myocardial function and structure, thus providing additional evidence on whether cardiac MRI is the optimal screening tool for the diagnosis of chemo or radiation therapy-induced cardiotoxicity, given its advantages in terms of quantitative assessment.

Recently, significant attention has been focused on the field of cardio-oncology. Many influential clinical guidelines for cardiovascular toxicity have been developed [8, 10, 16–19]. The current diagnostic criteria are based on left ventricular functional changes [17]. However, chemotherapy-induced cardiotoxicity is considered a continuum that begins with subclinical myocardial cell injury, leading to an early, asymptomatic decline in LVEF, which can eventually develop into symptomatic heart failure [12, 20]. LVEF can be used to detect myocardial damage only after functional impairment has already occurred.

Cardiac MRI is the gold standard for the evaluation of ventricular volumes and function, with greater intra- and inter-observer reproducibility than other methods, and quantitative cardiac MRI may play an important role in the cardiac evaluation and risk stratifications of cancer patients [11, 21]. Recently, many papers have been published that show the usefulness of MRI-obtained parameters such as ventricular volume, function, mass, and strain in diagnosing CTRCD [11, 22–26]. The most important capability of MRI is tissue characterization with quantitative MR parameters. Tissue characterization by cardiac MRI can help identify early myocardial injuries that are not included in the current diagnostic criteria [11, 21, 27].

Breast cancer therapy involves many adjuvant therapies in addition to surgery, most of which cause heart-related problems. Anthracycline, used as a primary chemotherapy agent in breast cancer, is a well-known cardiotoxic agent [8, 28], with the incidence of cardiotoxicity ranging from 4 to > 36% in patients receiving 500–550 mg/m^2^.

Other conventional chemotherapy agents used in breast cancer treatment, including cyclophosphamide and taxanes, also induce myocardial dysfunction [8]. The recently adopted targeted therapy agent trastuzumab, which improves outcomes of patients with HER2-positive breast cancer, is also notoriously associated with treatment-related cardiotoxicity [8].

Radiation therapy, another major treatment for breast cancer, induces myocardial fibrosis, microcirculatory injury, and myocardial infarction. High-dose radiation exposure is used on the thorax in the adjuvant setting after breast surgery [29].

Therefore, cardiac evaluation during breast cancer treatment is clinically very important. This study is based on the observation that it is very efficient to perform an additional myocardium examination during the routine surveillance of breast cancer. Indeed, the separate assessment of cardiac function using echocardiography or MUGA on a regular basis during surveillance of breast cancer treatment is somewhat cumbersome. Moreover, serial MUGA evaluations may cause significant radiation exposure. For these reasons, we set up a short-term imaging protocol for the evaluation of myocardial tissue and myocardial dysfunction, and we tested the feasibility of cardiac imaging using a breast coil in the preliminary study.

The limitation of this study is that it is a single-center prospective registry study. Although blood biomarkers such as NT proBNP or troponins have been demonstrated to be good early markers of cardiotoxicity, they are not included in the study protocol.

In conclusion, the simultaneous evaluation of breast cancer and cardiac function during treatment can potentially be very effective in terms of cost and time. Moreover, it may help the early detection of cardiotoxicity during cancer therapy, while quantitative imaging methods may provide additional value for the surveillance of patients at risk of CTRCD.

## Data Availability

Not applicable.

## Abbreviations

MRI: Magnetic resonance imaging
LV: Left ventricular
RV: Right ventricle
EF: Ejection fraction
LVEF: Left ventricular ejection fraction
ECV: Extracellular volume fraction
MACE: Major adverse cardiac event
MUGA: Multigated acquisition
CTRCD: Cancer therapy-related cardiac dysfunction
eGFR: Estimated glomerular filtration rate
STIR: Stimulated inversion recovery
DCE: Dynamic postcontrast enhanced
Hct: Hematocrit
LVEDV, RVEDV: LV or RV end-diastolic volume
LVESV, RVESV: LV or RV end-systolic volume
ROI: Region of interest
AUC: Area under the curve
ICC: Intraclass correlation coefficient
